# The Effect of a 12-Week Moderate Intensity Interval Training Program on the Antioxidant Defense Capability and Lipid Profile in Men Smoking Cigarettes or Hookah: A Cohort Study

**DOI:** 10.1155/2015/639369

**Published:** 2015-01-14

**Authors:** Abdessalem Koubaa, Moez Triki, Hajer Trabelsi, Hamza Baati, Zouhair Sahnoun, Ahmed Hakim

**Affiliations:** ^1^Laboratory of Pharmacology, Sfax Medicine Faculty SMF, Avenue Majida Boulila, 3029 Sfax, Tunisia; ^2^Laboratory of Cardio-Circulatory, Respiratory, and Hormonal Adaptations to Muscular Exercise, Ibn El Jazzar Medicine Faculty, Avenue Mohamed Karoui, 4002 Sousse, Tunisia; ^3^Research Unit (EM2S), Sfax Institute of Sport and Physical Education, Airport Road, P.O. Box 384, 3000 Sfax, Tunisia

## Abstract

*Aim*. To examine the impact of interval training program on the antioxidant defense capability and lipid profile in men smoking cigarettes or hookah unable or unwilling to quit smoking. *Methods*. Thirty-five participants performed an interval training (2 : 1 work : rest ratio) 3 times a week for 12 weeks at an intensity of 70% of VO_2_max. All subjects were subjected to a biochemical test session before and after the training program. *Results*. The increase of total antioxidant status (TAS), glutathione peroxidase (GPx), and *α*-tocopherol, is significant only for cigarette smokers (CS) and hookah smokers (HS) groups. The decrease of malondialdehyde (MDA) and the increase of glutathione reductase (GR) are more pronounced in smokers groups compared to those of nonsmokers (NS). Superoxide dismutase (SOD) increases in NS, CS, and HS groups by 10.1%, 19.5%, and 13.3%, respectively (*P* < 0.001). Likewise, a significant improvement of high-density lipoprotein cholesterol (HDL-C) and TC/HDL-C ratio was observed in CS and HS groups (*P* < 0.05). *Conclusion*. Although the interval training program does not have a significant effect on blood lipid levels, it seems to be very beneficial in the defense and prevention programs of oxidative stress.

## 1. Introduction

Smoking contains an abundance of free radicals and prooxidant species known to negatively influence human health [1]. Increased production of free radicals from tobacco is recognized because of the more than 4,000 chemical substances found in tobacco [2]. Therefore, ill-health related to smoking may be linked to increased production of free radicals.

Cigarettes and hookah consumption have risks of addiction, illness, or death. Koubaa et al. [3] evaluated the consumption harms of hookah in relation to cigarettes among sedentary adults, by measuring biochemical and cardiorespiratory parameters. This study reinforces the evidence that the hookah consumption is associated with exposure to toxic substances and has adverse effects on the cardiorespiratory and metabolic quality and produces in some cases the same effects as cigarettes. A lot of evidence suggests that hookah has harmful effects similar to cigarettes smoking.

Oxidative stress, having a strong association with many disease states including cardiovascular diseases (CVD), has recently been presented [4, 5]. Oxidative stress describes a state of physiological stress in the body that arises from exposure to high levels of reactive oxygen species (ROS) to an extent that overwhelms the antioxidant defense system [6]. Cigarette smoking exacerbates ROS formation [7], evidenced by the increase in oxidative stress biomarkers in smokers compared with nonsmokers [8, 9]. Free radicals can interact with molecules in the body and damage various cell components such as DNA, protein, and lipids, giving rise to various disease states [10]. Halliwell [11] suggests that oxidative damage of cell components has been implicated in the pathogenesis of a wide variety of diseases, most notably heart disease and cancer. A growing body of evidence suggests that oxidation of low-density lipoprotein (LDL) is believed to promote atherosclerosis [12]. Recent studies suggest that free radicals may be involved in the development of pulmonary disorders such as asthma [13]. Other major pathologies that may involve free radicals include neurological disorders and cataracts [14].

In preventing or slowing the progression of both heart disease and some forms of cancer, previous studies suggest that antioxidants may play a pivotal role [15, 16], and a low blood level of antioxidants is related with increased cancer risk [16]. Previous surveys indicate that smokers have oxidative stress levels of rest that are higher compared to nonsmokers and this can be explained, in part, by reduced blood antioxidant capacity [17, 18]. Cigarette-smoke-induced oxidative stress poses a significant human health concern, especially as related to cardiovascular disease [19].

So far, interventions to reduce harmful effects of tobacco have focused on the use of innovative tobacco products and the reduction of tobacco consumption and pharmaceutical drugs. Therefore, there is a need to expand the range of potentially effective strategies to reduce the harmful effects of tobacco in smokers who are unable or unwilling to quit. Our objective is to examine whether physical activity has the potential to become such a strategy so that it can strengthen the antioxidant defense system and improve the lipid profile and therefore to overcome this known unrest initiated by tobacco.

The lipid is an integral part of the search for the factors of cardiovascular risk. Previous studies have shown that smoking cigarettes or hookah produces significant increase in LDL-C and is associated with a high TG concentration and a reduced HDL-C [20, 21]. Other published reports suggest that cigarette smoking has been found to alter the lipoprotein levels [22]. Also, the effects of elevated lipid levels and changes in lipoprotein among cigarette smokers were demonstrated earlier [23–25]. In contrast, on the ability to control and to address this disturbance, several prior studies showed the physical activity effect on lipid profile improvement. The results of Thune et al.'s study [26] showed a dose-dependent inverse relationship between physical activity and lipid parameters. They found a significant decrease in total cholesterol (TC), triglycerides, and TC/HDL ratio and, thereby, an increase of HDL-C.

Most studies consider that continuous training is beneficial for maintaining cardiovascular health. However, the exercise with intervals can be more effective [27]. They suggested that the intermittent exercise of moderate intensity is beneficial for cardiovascular condition.

DeBusk et al. [28] found that subjects who exercised intermittent training sessions showed a gain of fitness and improvement in their blood lipid profile. Previous studies have suggested that short intermittent periods of walking improve plasma lipoproteins and blood pressure compared to a continuous training session [29]. In addition Macfarlane et al. [30] suggested that training periods with intermittent exercise led to a significant improvement in cardiovascular fitness. Physical activity reduces, therefore, the risk of cardiovascular disease and prevents strokes [29].

Exercise may have the potential to mitigate some of the negative consequences of smoking cessation. Bock et al. [31] and Marcus et al. [32, 33] have conducted a number of studies to examine this issue and they suggested that vigorous exercise (60–85% of heart rate reserve) can be a useful aid to quit smoking. To the best of our knowledge, there is a lack of empirical evidence to document if there are beneficial effects on the physiological symptoms of people who continue smoking.

Anaerobic training has been shown capable of increasing muscle antioxidants (SOD, GPx, and GR) and strengthening the body against new oxidative attack [34, 35]. Some studies have reported a decrease in antioxidant defenses of the body, which could be due, in part, to an excessive production of RL by cumulative effect of anaerobic exercise [36, 37] or to a food intake, least rich in antioxidants during the intervention period of participants [38, 39]. Within certain limits, it seems possible that anaerobic training can result in attenuation of rest oxidative stress similar to the aerobic training.

Although most training programs have involved continuous aerobic and anaerobic training sessions of long durations, recent work demonstrated that interval training can stimulate similar, if not superior, changes in cardiovascular function in both healthy [40] and clinical populations [41, 42]. The potential stimulus provided by intermittent training at a high intensity, yet often at a reduced total exercise volume, offers an efficacious alternative to continuous training.

Training benefits are optimized when programs are planned to meet the individual capacities of the participants. Therefore respiratory capacity must be taken into account in order to meet individual needs in training of sedentary smoker participants. The interest in assessing intermittent training was based upon previous experience of the acceptance of this training type in clinical practice [43] and because interval exercise resembles the daily life activity pattern in smokers more than continuous exercise.

Interval exercise seems to be an important factor for improving aerobic capacity, cardiovascular function, and quality of life in smoker participants that may have important implications in antioxidant capacity and serum lipid concentrations.

The aim of our study is to examine the effects of intermittent training on blood lipids and antioxidant defense capacity in sedentary male smokers and to check the difference of these individual effects of training among cigarette smokers compared to hookah ones.

## 2. Subjects and Methods

Our population was composed of adults matched in gender and age from the same ethnicity and socioeconomic environment. In fact, thirty-five sedentary male smokers and nonsmokers in good health from the general community of Tunisia (North Africa), which belongs to the public function (profession does not require physical exertion), volunteered to participate in this study and were recruited within the Pharmacology Laboratory of the Faculty of Medicine, University of Sfax, Tunisia. The anthropometric and physical characteristics of participants are shown in Table 1.

Participants were admitted to the training program after approval of cardiologist physician. They were normolipidemic (fasting triglycerides <1.7 mmol/L) and nonobese. No subject used nutritional supplements or medications. Presence of any kind of disease (based on history, medical examination, and exercise stress testing) and involvement in regular physical activity or exercise program during the previous 12 month were also exclusion criteria. On the basis of these criteria, 7 subjects from 42 were excluded. Finally, 35 subjects were included in subsequent analysis.

After receiving a complete verbal description of protocol, risks, and benefits of the study, subjects provided written consent to an experimental protocol approved by the Research Ethics Committee of the Faculty of Medicine, University of Sfax, Tunisia.

Cigarette and hookah smokers were recruited according to the number of cigarettes and hookah per day and their career period. We consider cigarette smokers as all subjects with consumption greater or equal to 10 pack-years (PY) and an average score of tobacco dependence of 7.33 ± 1.67, measured by the Fagerström nicotine dependence test [44]. In the absence of specific international codification, we quantified hookah consumption, as in the study of Kiter et al. [45], in NA and kg of cumulative tobacco. The tobacco used in a hookah weighs between 10 and 25 grams [46]. In fact, regular hookah smoking subjects are those having consumption greater than or equal to 5 hookah-years (HY) [47].

**Figure 1:**
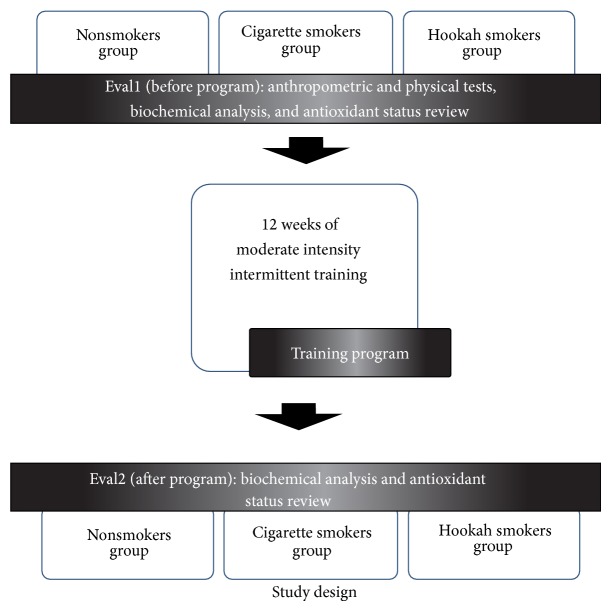


They were divided into three equal groups, and they performed an interval training 3 times a week for 12 weeks at an intensity of 70% of VO_2_max: a cigarette smokers' group (CS) (*n* = 11), a hookah smokers' group (HS) (*n* = 12), and other nonsmokers' group (NS) (*n* = 12). All subjects were subjected to a biochemical and metabolic test session before and after the training program. The session includes anthropometric and physical tests, a biochemical analysis, and antioxidant status review. All these measurements were performed by the same examiner to avoid methodological uncertainties. An exercise testing was performed before program to quantify the training individual loads (see Figure 1).

Body weight was measured to the nearest 100 grams using a calibrated electronic scale (Tanita TBF.350 model), and height was measured to the nearest 1 mm with a fixed stadiometer.

Analyses were performed in the Laboratory of Pharmacology, Faculty of Medicine of Sfax. Smokers were instructed to refrain from smoking for a one-hour period prior to reporting to the lab as suggested by Dietrich et al. [48].

Venous blood samples (antecubital vein) were taken in dry tubes under basal conditions (8 a.m.). After centrifugation, the sera were frozen at −80°C until analysis. Total cholesterol (TC), triglycerides (TG), and high-density lipoprotein cholesterol (HDL-C) were measured in all subjects after a 12-hour overnight fast using the standardized techniques described by Wegge et al. [49]. Low-density lipoprotein cholesterol (LDL-C) was calculated with the Friedewald formula [50]:

(1) $$\begin{array}{c} \left[\text{LDL}=\text{TC}-\text{HDL}-\left(\frac{\text{TG}}{2},18\right)\right]. \end{array}$$

Plasma concentrations of SOD, GPx, and the TAS were measured spectrophotometrically using a spectrophotometer type DU-640 (Beckman Instruments, Inc., CA, USA) and the dosage kits of TAS and antioxidant enzymes (SOD and GPx) were taken from Randox Laboratories.

VO_2_max and max heart rate measurements during exercise were examined at the triangular test with speedwalk (COSMED Pulmonary-Function Equipment, 37 Via dei Piani di Monte Savello, Rome, Italy). This dynamic and maximum test, until fatigue, consists in increasing the speed of 1 km/h every 2 min, after warm-up for 5 min with a 6 km/h speed. Heart rate and VO_2_ during the test were measured using an analyzer (version 1.2 PRO Fit mate COSMED).

Subjects of the three groups underwent an intermittent training program that consists of 3 sessions per week lasting 30 min, during a 3-month period. The intensity of the exercise was controlled by time and traveled distance. All warm-ups before training should be between 50% and 60% of maximum heart rate for a period of about 10 minutes.

The subjects participated in training sessions at evening using intermittent exercises. It is to run periods of 2 min race interspersed with recovery periods of one minute (2 : 1 work: rest ratio). The exercise intensity was 70% of VO_2_max. The load increase during the training period was provided by the repetition number. The training load was insured by time and traveled distance and controlled by beep sounds: *T* = *d*/*V* (*T*: the time between two studs; *d*: distance between two studs; and *V*: proposed speed). All participants did their best and they successfully completed the training period and no recorded absences during all training sessions. Furthermore, we have verified that there was no involvement in physical activity or exercise program elsewhere during the 12-week training period.

All statistical tests were processed using STATISTICA software (StatSoft, France). The data are expressed as mean ± SD (standard deviation). After normality verification with the Shapiro-Wilk *w* test and homogeneity of variances with Levene's test, parametric tests were performed. One-way ANOVA was used to indicate intergroup differences in the baseline subjects' characteristics. Inter- and intragroup comparisons of the variables were made by two-way ANOVA (group versus training) with repeated measurements. Least significant difference (LSD) post hoc analysis was used to identify significant group differences that were indicated by one-way and two-way ANOVA. Effect sizes were calculated as partial eta-squared (*η*_*p*_^2^) to estimate the meaningfulness of significant findings. A probability level of 0.05 was selected as the criterion for statistical significance.

## 3. Results

Through the anthropometric and physical measurements of participants (Table 1), no statistical differences were noted between the 3 groups for the four above variables: age, height, weight, and BMI (*P* > 0.05).

The LSD post hoc test for means comparisons allowed us to conclude that the two groups CS and HS have resting heart rate and resting SBP similar to and significantly higher than those of nonsmokers (*P* < 0.001). Furthermore cigarette smokers have developed a VO_2_max statistically higher than hookah smokers.

Before our training program, most of the antioxidant blood concentrations were similar in all of subjects smoking cigarette and hookah and different to those of nonsmokers (Table 2). Defense capability of SOD and the oxidative stress indicator level (MDA) of hookah smokers were significantly superior to those of cigarette smokers (*P* < 0.05). There were no significant differences in GPx and TAS concentrations of the three groups' subjects.

The differences in the antioxidants values (Δ) of the three groups of our population before versus after program are summarized in Table 3. In CS and HS groups, the GPx increase is significant; it is of the order of 6.5 ± 5.04 (U*·*gHg^−1^) and 7.23 ± 4.79 (U*·*gHg^−1^), respectively (*P* < 0.01), while it is only 2.8 ± 5.19 (U*·*gHg^−1^) in NS group (*P* > 0.05).

**Figure 2:**
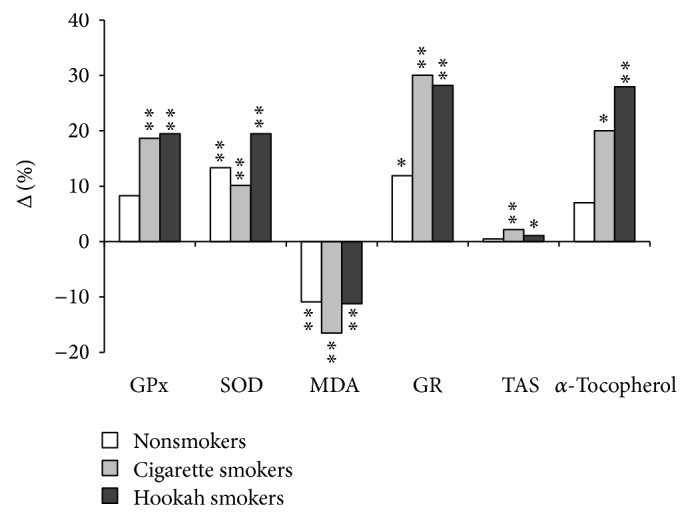
Antioxidants improvement rate in percentage of the three groups after training program. GPx: glutathione peroxidase; SOD: superoxide dismutase; MDA: malondialdehyde; GR: glutathione reductase; TAS: total antioxidant status; ^*^ *P* < 0.05; ^**^ *P* < 0.01; ^***^ *P* < 0.001.

Similarly, the MDA decrease is more pronounced in CS and HS groups compared to that of NS. It is, respectively, −0.25 ± 0.231 (*μ*mol*·*l^−1^), −0.186 ± 0.09 (*μ*mol*·*l^−1^), and −0.158 ± 0.177 (*μ*mol*·*l^−1^) (*P* < 0.01, *P* < 0.01, and *P* < 0.05, resp.). The GR increase follows the same pattern. It is 2.49 ± 0.88 (U*·*gHg^−1^) in CS and 2.44 ± 1.25 (U*·*gHg^−1^) in HS while it is only 1.24 ± 1.7 (U*·*gHg^−1^) in the NS group (0.05 < *P* < 0.001). Concerning blood levels of TAS and *α*-tocopherol, the increase is significant only in CS and HS subjects. This is, respectively, 2.2% (*P* < 0.01) and 20% (*P* < 0.05) in CS group and 1.1% (*P* < 0.05) and 28% (*P* < 0.01), respectively, in HS group (Figure 2). Finally, subjects in CS, HS, and NS groups showed SOD values increased. The increase is, respectively, 10.1%, 19.5%, and 13.3% (*P* < 0.01).

Before training, blood concentrations of TC and LDL-C were significantly similar in the three groups. Concerning HDL-C/TG and TC/HDL-C reports and lipid concentrations in HDL-C and TG, Table 4 shows significant differences between smokers and nonsmokers (*P* < 0.001). Our results uncover a single significant difference between cigarette smokers and hookah smokers and it is at the TC (*P* = 0.035).

Our intermittent training program induced a concentrations' decrease of all parameters, but it is only significant at the level of CL/HDL-C ratio of both groups CS and HS. This is, respectively, 0.2 ± 0.28 and 0.2 ± 0.24 (*P* < 0.01). Further, the HDL-C increase was significant after our program in both groups CS and HS. It is, respectively, 0.04 ± 0.06 (mmol*·*l^−1^) and 0.03 ± 0.05 (mmol*·*l^−1^) (*P* < 0.05). All data are presented in Table 5.

**Figure 3:**
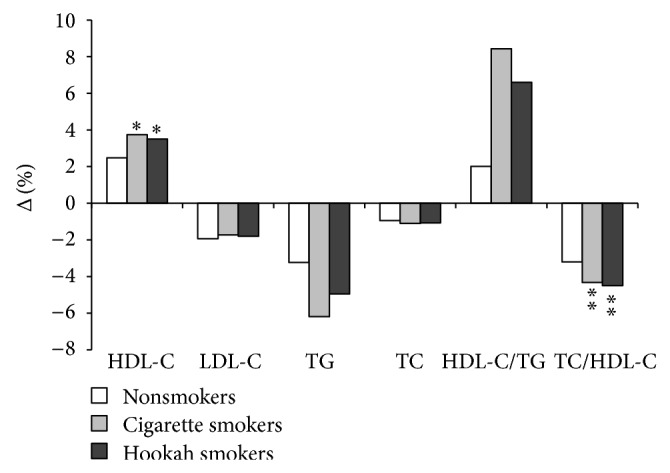
Lipid improvement rate in percentage of the three groups after training program. HDL-C: high-density lipoprotein cholesterol; LDL-C: low-density lipoprotein cholesterol; TC: total cholesterol; TG: triglycerides; ^*^ *P* < 0.05; ^**^ *P* < 0.01.

In the three groups, Figure 3 showed a reduction of TC, TG, and LDL-C and an increase in HDL-C/TG ratio. However, these parameters improvement was not significant (*P* > 0.05) and was lower in NS subjects.

## 4. Discussion

This study indicates that either cigarette or hookah smokers have low basal antioxidant capacity and have, therefore, important levels of oxidative stress compared to nonsmokers (Table 2). These data suggest that smoking can independently promote a negative change according to the number of years of smoking. Many researchers have reported high levels of oxidative stress in smokers compared to nonsmokers [17, 18].

Indeed, several studies have examined, using different protocols, the effect of physical exercise or training on antioxidant status. For this, we chose to determine the intermittent training's contribution to the antioxidant defense capacity in sedentary adult smokers.

According to Bloomer et al. [6, 51], anaerobic exercises could help to increase rest antioxidant defenses and reduce the oxidants production during and after exercise.

In our study, the antioxidant defenses were increased at the main effect of intermittent training. These results are consistent with Finaud's study which in turn showed an increased antioxidant capacity [52].

At the end of the training period proposed to our subjects, changes in their antioxidant capacity were illustrated. They are characterized by an increase of superoxide dismutase (SOD) and glutathione reductase (GR) and a decrease of malondialdehyde (MDA) retaining the glutathione peroxidase (GPx) and *α*-tocopherol for nonsmokers. Our findings are concomitant with the results of previous studies [6, 53, 54]. This increase is similar to that found by Bloomer et al. [6, 53].

In addition, the *α*-tocopherol improvement recorded in our study varies by groups. It is greater in subjects of CS and HS compared to those of NS. These results join those published by Cuevas et al. [34] and differ from those reported by Pialoux et al. [37].

The studies dealing with the effect of training on the antioxidants of smoker subjects showed varying changes [36, 37]. This divergence could be explained, in part, by the diversity of protocols implemented (training methods, protocol duration, age of participants, smoking duration, etc.) and the individual responses of each subject.

Because we found no statistical difference between the two smoking groups for all variables after training, we have no reason to believe that, in this regard, a group was affected more than another group.

It should be noted that, despite the absence of a dietary survey, antioxidant improvement was significant in most cases for the three participating groups and was more pronounced in smokers versus nonsmokers. It seems that this training method was sufficient to improving antioxidant defense capacity and mitigating oxidative stress in smokers and nonsmokers. Exercises of different intensity and duration may be ineffective to overcome this known unrest initiated by smoking.

Several studies examining the intermittent training effect on lipid profile showed a controversial effect [55, 56], but, to our knowledge, it was not yet shown if this training method would have more favorable effects on blood lipid profiles, especially in adult smokers. Indeed, our intermittent training program has no effect on lipid metabolism of nonsmoking subjects. A wide literature showed little change in the control groups when they participated in training protocols [57, 58].

The broad consensus in the literature [58–60] and the findings of this study have shown that intermittent training has no significant effects on the TC rate and LDL-C levels. However, several other studies have shown that intermittent training may actually decrease the TC rate [55, 61] and LDL-C and had no effect on HDL-C levels [60, 62, 63]. While in some cases [55, 58, 61, 64], including those of the present study, intermittent training was found to increase HDL-C, a possible explanation is that the studies that have found decreased HDL-C levels, used, perhaps, samples with lower baseline HDL-C levels and shorter studies periods.

As in the case of this study, which showed a decrease in TC/HDL-C ratio for CS and HS groups, several other studies have shown also a decrease in this ratio [55, 65]. Moreover, intermittent exercise training had no effect on triglycerides and LDL-C/TG ratio. These results confirm the Frey MA findings [62].

In short, our study showed that the intermittent training method was not associated with favorable changes in lipid and lipoprotein levels in all smoker subjects of both cigarette and hookah; therefore, it cannot prevent the progress of cardiovascular diseases.

Finally, our study proposed an additional demonstration concerning intermittent training method which could be prescribed and recommended in smoker subjects. This training protocol improves blood antioxidants and therefore reduces oxidative stress. It has no significant effect on lipids and lipoprotein profile, except HDL-C and CL/HDL-C ratio of smoker participants. This training method seems to have a more transparent effect by combining to a dietary follow-up.

## 5. Conclusion

The present study demonstrates that training with intermittent exercises improves blood antioxidants. Intensity, recovery, and training volumes have been closely monitored to demonstrate the intermittent exercise importance to reduce oxidative stress in cigarette and hookah smokers. Physical training with intermittent exercises seems to be very beneficial in the prevention of oxidative stress. These results could have important implications in defense and prevention programs. Although our study using interval exercises does not have a significant effect on blood lipid levels, other studies using other training methods will be needed to advance our conclusions. We believe that the continuous exercise training programs could affect more favorably the lipid and lipoprotein profile of smoker subjects than training programs with intermittent exercises.

*Practical Implications*

- Smokers before training present higher oxidative stress compared with nonsmokers.
- Improvement in antioxidant system capacity is significantly higher in smokers than in nonsmokers.
- Smokers before training present worst lipid profile compared with nonsmokers (HDL-C, TG, HDL/TG ratio, and TC/HDL-C).
- Significant improvements were obtained only in HDL-C and TC/HDL-C ratio in smokers.
- People who are unable to quit smoking could focus on improving leisure time physical activity (by intermittent exercises) to reduce the harm caused by smoking.

*Limitations of the Study.* One limitation of the study is that diet during the training period was not controlled. However, study requires that participants follow the same diet in the 3 days preceding each blood sampling and during the training period.

The lack of a control group (smokers follow the same daily activity during the protocol period) may be considered a limitation of the present study.

Finally, our relatively small sample size could have limited our ability to detect group differences in our chosen markers. This is indeed a limitation of this work and should be considered relative to our findings.

## Figures and Tables

**Table 1:** Anthropometric and physical characteristics of participants (mean ± SD).

Parameters	Nonsmokers	Cigarette smokers	Hookah smokers	ANOVA
Age (yrs)	44.5 ± 1.3	45.5 ± 1.7	44 ± 1.7	*F*(2; 33) = 0.84; *P* = 0.47; *η*_*P*_^2^ = 0.17
Height (cm)	173.8 ± 1.3	175.8 ± 2.8	173 ± 1.6	*F*(2; 33) = 0.91; *P* = 0.43; *η*_*P*_^2^ = 0.15
Weight (kg)	71.4 ± 2.2	72.7 ± 3	69.6 ± 2.2	*F*(2; 33) = 1.65; *P* = 0.24; *η*_*P*_^2^ = 0.25
BMI (kg·m^−2^)	25.1 ± 1.1	24.4 ± 1	24.7 ± 1.2	*F*(2; 33) = 1.2; *P* = 0.31; *η*_*P*_^2^ = 0.07
Resting heart rate (bpm)	78 ± 4	91 ± 2^***^	93 ± 4^***^	*F*(2; 33) = 66.52; *P* < 0.001; *η*_*P*_^2^ = 0.80
Resting SBP (mmHg)	131 ± 3	138 ± 3^***^	138 ± 3^***^	*F*(2; 33) = 27.91; *P* < 0.001; *η*_*P*_^2^ = 0.63
Resting DBP (mmHg)	85 ± 6	87 ± 5	86 ± 4	*F*(2; 33) = 0.48; *P* = 0.62; *η*_*P*_^2^ = 0.03
VO_2_max (mL·min·kg^−1^)	37.5 ± 1.6	38.9 ± 2.5	36.6 ± 1.2^##^	*F*(2; 33) = 4.79; *P* = 0.015; *η*_*P*_^2^ = 0.22

BMI: body mass index; resting SBP: systolic blood pressure at rest; resting DBP: diastolic blood pressure at rest; VO_2_max = maximum oxygen uptake; ^***^significant differences compared to nonsmokers at *P* < 0.001; ^##^significant differences compared to cigarette smokers at *P* < 0.01.

**Table 2:** Antioxidant concentrations before training (mean ± SD).

Parameters	Nonsmokers	Cigarette smokers	Hookah smokers	ANOVA
GPx (U·gHg^−1^)	37.12 ± 2.6	34.84 ± 4.31	33.84 ± 5.07	*F*(2; 33) = 2.01; *P* = 0.15; *η*_*P*_^2^ = 0.11
SOD (U·gHg^−1^)	1651.3 ± 105.2	1432.1 ± 171.2^***^	1545.1 ± 105.9^*^	*F*(2; 33) = 8.1; *P* = 0.0014; *η*_*P*_^2^ = 0.33
MDA (*μ*mol·L^−1^)	1.454 ± 0.17	1.517 ± 0.095	1.663 ± 0.111^∗∗∗#^	*F*(2; 33) = 8.1; *P* = 0.0014; *η*_*P*_^2^ = 0.33
GR (U·gHg^−1^)	10.46 ± 2.01	8.3 ± 1.55^**^	8.66 ± 1.6^*^	*F*(2; 33) = 5.43; *P* = 0.009; *η*_*P*_^2^ = 0.25
TAS (mmol·L^−1^)	1.8 ± 0.02	1.78 ± 0.01	1.79 ± 0.02	*F*(2; 33) = 2.7; *P* = 0.082; *η*_*P*_^2^ = 0.14
*α*-tocopherol (*μ*mol)	6.78 ± 0.95	5.24 ± 0.88^***^	5.19 ± 0.88^***^	*F*(2; 33) = 12.42; *P* < 0.001; *η*_*P*_^2^ = 0.43

GPx: glutathione peroxidase; SOD: superoxide dismutase; MDA: malondialdehyde; GR: glutathione reductase; TAS: total antioxidant status; ^∗,∗∗,∗∗∗^significant differences compared to nonsmokers at *P* < 0.05, *P* < 0.01, and *P* < 0.001, respectively; ^#^significant differences compared to cigarette smokers at *P* < 0.05.

**Table 3:** Antioxidants' improvement rate (Δ) of the three groups after 12 weeks of intermittent training.

Parameters	(Δ): mean ± SD			Results signification		
	NS	CS	HS	NS	CS	HS
GPx (U·gHg^−1^)	2.8 ± 5.19	6.5 ± 5.04	7.23 ± 4.79	ns	‡	‡
SOD (U·gHg^−1^)	190.8 ± 129.2	167.4 ± 191.9	300.8 ± 126.9	‡	‡	‡
MDA (*μ*mol·L^−1^)	−0.16 ± 0.18	−0.25 ± 0.23	−0.19 ± 0.09	†	‡	‡
GR (U·gHg^−1^)	1.24 ± 1.7	2.49 ± 0.88^*^	2.44 ± 1.25^*^	†	‡	‡
TAS (mmol·L^−1^)	0.01 ± 0.03	0.04 ± 0.02^*^	0.02 ± 0.03	ns	‡	†
*α*-Tocopherol (*μ*mol)	0.48 ± 0.78	1.05 ± 1.53	1.45 ± 0.8	ns	†	‡

(Δ): mean change; NS: nonsmokers; CS: cigarette smokers; HS: hookah smokers; ns: nonsignificant; GPx: glutathione peroxidase; SOD: superoxide dismutase; MDA: malondialdehyde; GR: glutathione reductase; TAS: total antioxidant status; ^*^significant differences compared to nonsmokers at *P* < 0.05; †, ‡: significant difference before versus after training program at *P* < 0.05 and *P* < 0.01, respectively.

**Table 4:** Blood lipid levels of smokers and nonsmokers before training program (mean ± SD).

Parameters	Nonsmokers	Cigarette smokers	Hookah smokers	ANOVA
HDL-C (mmol·L^−1^)	1.12 ± 0.12	0.99 ± 0.04^***^	0.97 ± 0.05^***^	*F*(2; 33) = 12.19; *P* < 0.001; *η*_*P*_^2^ = 0.42
LDL-C (mmol·L^−1^)	2.89 ± 0.22	2.9 ± 0.1	2.75 ± 0.17	*F*(2; 33) = 2.75; *P* = 0.079; *η*_*P*_^2^ = 0.14
TG (mmol·L^−1^)	0.9 (0.2)	1.28 (0.22)^***^	1.38 ± 0.32^***^	*F*(2; 33) = 12.51; *P* < 0.001; *η*_*P*_^2^ = 0.43
TC (mmol·L^−1^)	4.42 ± 0.12	4.48 ± 0.09	4.36 ± 0.11^#^	*F*(2; 33) = 3.73; *P* = 0.035; *η*_*P*_^2^ = 0.18
HDL-C/TRIG	1.29 ± 0.32	0.8 ± 0.15^***^	0.74 ± 0.15^***^	*F*(2; 33) = 22.45; *P* < 0.001; *η*_*P*_^2^ = 0.58
TC/HDL-C	4 ± 0.44	4.52 ± 0.18^***^	4.49 ± 0.22^***^	*F*(2; 33) = 11.09; *P* < 0.001; *η*_*P*_^2^ = 0.40

HDL-C: high-density lipoprotein cholesterol; LDL-C: low-density lipoprotein cholesterol; TC: total cholesterol; TG: triglycerides; ^***^significant difference compared with nonsmokers at *P* < 0.001; ^#^significant difference compared with cigarettes smokers at *P* = 0.035.

**Table 5:** Lipid improvement rate (Δ) of the three groups after 12 weeks of intermittent training.

Parameters	(Δ): mean ± SD			Results signification		
	NS	CS	HS	NS	CS	HS
HDL-C (mmol·L^−1^)	0.03 ± 0.05	0.04 ± 0.06	0.03 ± 0.05	ns	†	†
LDL-C (mmol·L^−1^)	−0.06 ± 0.1	−0.05 ± 0.15	−0.05 ± 0.14	ns	ns	ns
TG (mmol·L^−1^)	−0.03 ± 0.12	−0.08 ± 0.13	−0.07 ± 0.16	ns	ns	ns
TC (mmol·L^−1^)	−0.04 ± 0.08	−0.05 ± 0.1	−0.05 ± 0.1	ns	ns	ns
HDL-C/TG	0.03 ± 0.22	0.07 ± 0.08	0.05 ± 0.07	ns	ns	ns
TC/HDL-C	−0.13 ± 0.16	−0.2 ± 0.28	−0.2 ± 0.24	ns	††	††

(Δ): mean change; NS: nonsmokers; CS: cigarette smokers; HS: hookah smokers; HDL-C: high-density lipoprotein cholesterol; LDL-C: low-density lipoprotein cholesterol; TC: total cholesterol; TG: triglycerides; ns: no significant difference (*P* > 0.05); †, ‡: significant difference before versus after training at *P* < 0.05, *P* < 0.01, respectively.
